# Publication subsidies: challenges and dilemmas facing South African researchers

**Published:** 2012-09

**Authors:** Angela J Woodiwiss

**Affiliations:** Cardiovascular Pathophysiology and Genomics Research Unit, School of Physiology, Faculty of Health Sciences, University of the Witwatersrand, Johannesburg, South Africa

**Keywords:** publication subsidy, Department of Higher Education and Training subsidy formulae, research outputs, higher education institutions

## Abstract

**Abstract:**

In an attempt to encourage and enhance research productivity in higher educational institutions, various systems have been introduced. Currently in South Africa, a government subsidy is granted to higher educational institutions in reward for research outputs (primarily journal publications and postgraduate student graduations). The purpose of this article is not to attack the current or past publication subsidy systems but rather to enlighten researchers, especially emerging researchers, on the benefits and risks of the publication subsidy systems and other systems used to encourage research outputs.

With this aim in mind, a comparison of the current versus the previous South African funding formulae will be made and the positive and negative impacts of these formulae (focusing primarily on the one currently in use) will be discussed in the light of international experiences using similar such approaches. In essence I wish to highlight the challenges and dilemmas faced by South African researchers and higher education institutions as they strive to find ways to increase research outputs while simultaneously sustaining or enhancing the quality and impact of these research outputs, in order to maintain and/or gain national and international recognition.

## Abstract

There is no doubt that the reputation, both national and international, of a higher educational institution is entrenched in its research profile, which depends to a major extent on its publications and the citations of these publications. Consequently, in aspiring to maintain and/or gain high-level profiles, all higher educational institutions strive to increase research outputs.

In South Africa, as in other countries, the Department of Higher Education and Training, in part to justify public expenditure (to provide greater accountability for the use of research funds), endorses these goals. Moreover, bearing in mind the academic adage ‘publish or perish’, individual researchers strive to maintain and/or increase their publication outputs. Indeed, in addition to a researcher’s reputation (both nationally and internationally) in a particular research field, successful attainment of grant funding as well as promotion are intimately linked to research outputs.

Like many countries around the world, South Africa has a system aimed to incentivise researchers and hence higher educational institutions to increase research outputs.1-8 The details of the South African systems (the previous system compared to the present system)8 will be discussed and then compared to those used by other institutions worldwide.1-7 There have been many criticisms raised against the various systems used to encourage research productivity within the different higher educational institutions worldwide. Indeed, a commentary on the politics of publication, published in *Nature* in 2003,9 incited a barrage of correspondence both in support of,6,10,11 and against12-14 various assessments of research outputs.

As it is advisable to avoid previous shortfalls in systems, especially those which may have long-lasting impacts on the reputation of a higher educational institution,3 it is important to consider these criticisms and their possible relevance in the context of the current South African system. Hence, the positive versus the negative impacts of the various systems used worldwide will be debated.

The system currently used to encourage research productivity by South African researchers at higher educational institutions is largely intended to be a financial reward-based system.8 Hence, the financial value (past and present) of research outputs will be presented. Comparisons will be made of the declining percentage of public expenditure on higher education as a percentage of the gross domestic product (GDP) in South Africa versus other countries in Africa,15 as well as worldwide.15-17 Moreover, as the intent of the South African system is to reward and hence encourage individual researchers,8 the differential distribution of the funds both between and within the different South African higher educational institutions will be described.

Bearing in mind the controversies raised against the various assessment systems used worldwide, the potential impact of possible declines in the financial value of research output units, and the potential inequity as a consequence of differential distribution practices within and between higher education institutions in South Africa, some proposals for the way forward will be presented.

## Comparison of subsidy formulae used in South Africa

In South Africa, government subsidies to higher educational institutions were previously (from 1987 to 2003) based on the South African post-secondary school (SAPSE) subsidy formula.8,15 The 1997 White Paper on Higher Education Transformation18 rejected this model and proposed its replacement with a new model aimed to bring equity and efficiency into the higher educational system.8 One of the main concerns with the SAPSE subsidy formula was the efficiency of outputs and outcomes, given that 50% of the formula was input driven.8

In 2004, the new funding formula (NFF) was implemented. As one of the ideas behind the NFF was to recognise the importance of research output,8 the NFF was intended to try and reverse the trend for a decline in research productivity. Indeed, research productivity by higher educational institutions was noted to have declined by 20% from 1997 to 2003.15

## The SAPSE subsidy formula

Using the SAPSE subsidy formula, the extent of subsidy payments to a higher educational institution was determined on a 50:50 weighting between inputs (predicted costs of student training and research) and outputs (student graduations, publications).16 The government subsidy was based on the determination of the actual costs of a reasonably efficient higher educational institution and decisions were made on which of these costs should be covered by government subsidies. The values attributed to the various cost units changed each year in accordance with inflation and changing cost patterns. For example, total actual government funding to higher educational institutions increased from R7 532 million in 2001/2 to R7 969 million in 2002/3.8

## The NFF subsidy formula

Using the NFF, the extent of subsidy consists of four block grants:8

• Teaching input grant (planned full-time equivalent student enrollments)
- Includes provision for research training
• Teaching output grant (non-research graduates produced)
- Includes provision for research training
• Research output grant (publications and postgraduates produced)
• Grant for other institutional factors (development).

Although the NFF was intended to be based on outputs to a greater extent than the SAPSE, this is not the case. If one considers the four block grants upon which the NFF is based, then indeed outputs contribute more than inputs. However, the percentage of the grant which makes up each of these blocks differs substantially. For example, in 2006 the percentages were: teaching input grant 65%; teaching output grant 15%; research output grant 13%; and grant for other institutional factors 7%.16 Furthermore, when one considers that both the teaching output grant and the research output grant include subsidies for poor performance, then a proportion of these already relatively small output grants are actually input grants.16 Hence, the NFF is to a larger extent than the SAPSE driven by input factors.19

The research output grant is based on units, whereby a publication in an accredited journal (see section titled ‘What constitutes an accredited journal’) is one unit; graduation of a student with an MSc by research is one unit; graduation of a student with an MSc by course work and short report is 0.5 unit (if the research component contributes 50%); and graduation of a student with a PhD is three units.8,20

A basis of the NFF is that the government first decides how much it can afford to spend on higher education and then it allocates funds according to its needs and priorities.8 Hence, in the NFF, it was initially proposed that the value per unit output was determined by the total funds available, divided by the total unit output. However, it was recognised that if the total funds available were set in advance, then an increase in total unit output would result in a decrease in the rand value per unit; hence the NFF would create a disincentive to increase research outputs.8

The proposal was therefore revised such that the value per unit is determined by:8

• setting a benchmark of weighted research output units per full-time permanent academic/research staff [weighted 1.2 for universities (recently increased to 1.4125) and 0.5 for universities of technology] (termed weighted research output)
• generating normative data of total outputs per year by relating above benchmarks per year to staff complements per year (termed normed research output)
• dividing the total government funds available for research outputs by normative total outputs.

In other words, each institution’s ‘delivery’ is determined by expressing their weighted research output as a percentage of their normed research output.20 The actual subsidy earned by an institution is equal to the institution’s weighted research output multiplied by the total government funds available for research outputs and divided by the average normed research output for all institutions.20

Of concern is that an institution with a delivery of less than 100% earns a developmental grant that is linearly related to the extent of their failure to deliver.20 Although, this idea of a developmental grant has its merits (encouraging institutions to develop a stronger research ethos), it is also a potential perverse incentive, in that institutes are rewarded more for doing less.

If the government determines the total of public funds that should be spent in a given year on higher education,8 then in essence the total government funds available for research outputs is set in advance. Therefore as research outputs increase, the value of the unit declines. Hence, although the NFF recognises the budget constraints of the country (it is driven by the availability of public resources for funding higher education),8 this formula is more likely to act as a disincentive than an incentive to increase research outputs.

In other words, given that the funds available for dispersal are finite, if all higher educational institutions increase their research outputs, then the monetary value of a research output unit will decline, a criticism which has been called the ‘zero-sum game’.21 Given that the basis of the NFF is to encourage future research output by means of rewards for past research outputs, a decrease in the monetary value of a research output unit is likely to create a disincentive to increase research outputs nationally.21

## What constitutes an accredited journal?

Worldwide most institutions base their research outputs on publications in journals that are listed in the Institute for Scientific Information’s (ISI) Science Citation Index, Social Sciences Citation Index and Arts and Humanities Citation Index.3 In South Africa, publications in those journals listed in the above three ISI indices qualify for subsidy, and in addition, publications in journals listed in the International Bibliography of Social Sciences (IBSS) index, or in the Department of Higher Education and Training-approved South African journals list qualify for subsidy.

To be eligible for inclusion in the list of South African journals accredited by the Department of Higher Education and Training, the purpose of the journal must be to disseminate research results, articles must be peer reviewed, journal contributions and distribution must be beyond a single institution, the journal must be published regularly, have an international standard serial number (ISSN) and have an editorial board. The Department of Higher Education and Training has also indicated that they plan to recognise three new journal databases for subsidy purposes in the future.

A pertinent issue is what constitutes a publication? For example, how are creative outputs such as artistic performances, paintings, concerts and novels recognised? Some higher educational institutions already have internal processes to take these outputs into account, although they are currently not recognised by the Department of Higher Education and Training. However, the Department of Higher Education and Training is at present wrestling with a procedure to recognise these outputs; hence this issue will not be discussed in this review. Further controversies, which are not discussed in this review, are what constitutes an accredited conference proceeding and the relative undervaluing of books and chapters in books within the South African subsidy formula.

## Comparisons of the South African system to encourage research productivity with systems used in higher educational institutions in other countries in the world

The system in South Africa is perceived to be unique in that it is intended to provide direct financial reward to individual researchers for their outputs.8,20 Although direct financial benefit to individuals may be intended, this is often not put into practice by the higher educational institutions, as will be discussed (see section titled ‘Distribution of Department of Higher Education and Training research subsidy within higher educational institutions in South Africa’).

In comparison, to South Africa’s ‘direct reward system’, other countries have various different strategies aimed to stimulate research outputs. For example, in the United States of America, successful leverage of research support from the federal government is based on the assessment of an individual’s research proposal, which incorporates details of his/her research track record.2

In the United Kingdom from 2014, obtaining government funding for research will be based on a system which places emphasis on an individual researcher’s outputs and the citation of his/her publications (the Research Excellence Framework, REF).4 The REF will replace the previous system used in the United Kingdom in the 1980s, 1990s and 2000s, namely the Research Assessment Exercise (RAE).7 The RAE, which was used to determine the amount of funding provided by the government to individual institutions, was based upon the credentials of a limited number of the most prominent researchers in each academic department within an institution.7

The South African system is not actually that unique. In Australia, government funds granted for research are based on research income, publication counts and higher degrees completed.3,5 Importantly, the Australian formula (similar to the South African NFF) is based solely on quantity, the impacts of which are discussed below in the section titled ‘Positive and negative impacts of various approaches used to encourage research productivity’. As occurs in South Africa, whether the funding goes to the individual researcher or the institution appears to differ between the higher degree institutions in Australia.3

In Spain, individual researchers are rewarded on the basis of publications; however a financial bonus is only awarded for publications in high-impact factor journals.6 A similar approach is used in Finland, except the institution rather than the individual researcher is the recipient of the financial benefits.1 The merits of rewarding individuals as opposed to institutions are discussed under the section titled ‘Financial reward of individuals versus institution’.

## Positive and negative impacts of various approaches used to encourage research productivity

In order to encourage research productivity, a measure of research outputs is mandatory. The common issue that arises is whether research outputs should be measured purely by counting (termed research output audits) or whether an element of research quality (for example citations or journal impact factor) should be taken into account. This debate is far from resolved, however a commentary on the politics of publication, published in *Nature* in 2003,9 incited a barrage of correspondence in which some interesting arguments both for,6,10,11 and against12-14 various measures of research productivity were raised.

## Quantity versus quality

A number of criticisms have been raised regarding the potential negative impact of research output audits on research quality.3,13 One of the major concerns is an encouragement of researchers to publish as many papers as possible (salami slicing)14,22 and to preferentially choose to publish in those journals which have the least rigorous review process.21

Indeed, in South Africa, although there was a 50 to 60% increase in the number of publications produced by universities from 1990 to 1994 and 2004 to 2008;23 only 57% of the publications that qualified for governmental subsidy in 2007 were published in internationally accredited journals.23 Although this percentage has increased to 64% in 2008, 66% in 2009 and 69% in 2010, this increase is partly attributed to an increase in the number of South African journals listed in the ISI and IBSS.24

However, more convincing evidence of an increase in quantity at the expense of quality is provided by data from a study conducted on higher educational institutions in Australia.3,13 In 1995, the formula by which the Australian government funds were distributed to higher educational institutions to support research activities was changed to incorporate publications.3,13 Consequently, researchers could calculate the financial value of a publication [A$761 (R6 664.31) to A$1089 (R9 536.71) between 1995 and 2000].13 As a consequence of increases in the total amounts to be distributed this value subsequently increased to A$3 000 (R26 271.94) in 2002.13

In her study, Butler13 allocated all journals from the ISI’s Science Citation Index into quartiles based on journal impacts calculated from five-year citation means. She then compared the Australian Universities’ share of publications within these quartiles prior to (1981–1985), with after (1996–2000) the incorporation of publications in the government formula. Although the share was stable within each quartile prior to the change in the funding formula, increases in the share occurred within each quartile after the change. However, of concern were the dramatically greater increases in the shares within the lower two quartiles (~100 and ~50%, respectively) compared to the increases in the shares in the higher two quartiles (only ~20% each).13 Hence, although the change in the funding policy resulted in noticeable improvements in the total number of publication outputs (an increase of 25%) at a time when academic staff numbers were stable,3 this increase was at the expense of quality.3,13

A study comparing the research outputs by two Australian universities lends further support to concerns of increased quantity at the expense of quality when rewards were based solely on the number of research outputs.3 In this study, the research outputs in terms of quantity and quality of two Australian universities, which were using different systems to enhance research outputs, were compared. In the university that provided financial incentives based only on quantity, although research outputs increased, there was a simultaneous decline in publications in high-impact factor journals (a measure of research quality).3 In the university that used a strategy in which the brightest young researchers were recruited and employed, the total number of publications increased as well as the number of publications in high-impact factor journals.3 These studies clearly demonstrate the negative impact of rewards based solely on the counting of the number of publications.

In comparison to the Australian approach, in Spain a system in which only high-quality publications were rewarded was instituted in 1989.6 To quote the Spanish parliamentary record, a bonus is awarded to individual researchers only for ‘…those articles of scientific worth in journals of recognized prestige in the field. …. In those disciplines for which international systems of quality of publications exist, reliance on these systems should be obligatory…’.6

Spanish research productivity doubled after the system of publication bonuses was passed into law.6 Indeed, twice as many publications were produced between 1991 and 1998 compared to between 1982 and 1990, and the number of Spanish publications in ISI databases was increased.6 These dramatic increments were not attributed to factors such as increased financial support, international collaboration or an increase in the number of staff.25

The consequences of this law were therefore that research productivity increased in both quantity and quality. Hence, the Spanish system of rewarding only high-quality publications appears to overcome the pitfalls of the Australian system, whereby rewarding research outputs irrespective of quality results in improved quantity at the expense of quality.

Further support of the use of a system based on rewarding researchers for publication in journals with a high impact factor was provided by Lomnicki10 in his discussion on the positive impact of such systems on research productivity in Germany and France. Lomnicki10 goes so far as to state that ‘abandonment of objective methods of science evaluation derived from the SCI’, would be most dangerous as ‘it would remove a tool for rewarding researchers who attempt to do good science and for eliminating those who do not’.

A lesson to be learnt from these previous experiences, is that in order to avoid possible ‘salami slicing’ of research (data that should constitute one publication is divided into many smaller publications),14,21,26 choosing in-house journals above international journals,21,27 and preferences for choosing journals which are perceived to be easier to publish in,21,26,27 the rewarding of research outputs as a means to encourage and enhance research outputs should be based on an assessment that includes the quality of publications.

The question therefore arises as to why high-quality publications are so important? The reason that some journals have higher impact is that they generate greater citations. In other words work published in higher impact journals is more highly cited than work published in lower impact journals. Hence, publications in high-quality journals are of greater value and have a superior impact on the scientific/research community.

However, the favouring of high-impact factor journals for the submission of manuscripts runs the risk of the journal choice becoming more important than the scientific message of the manuscript.28 Indeed, it has been suggested that authors wishing to have their work published in the journal *Nature* claim novelty in their work, which is not entirely true, in order to enter into the review process.12 During the course of the review such ‘false novelty’ is then identified and the manuscript is changed accordingly prior to publication.12 However, the result is that work is published in *Nature* that is no more novel than that published in high-quality specialised journals.12

Nevertheless, there are other important benefits of sending manuscripts to high-impact factor journals.11 Firstly, authors are more likely to receive meaningful feedback, as the top journals in each field are most likely to consult the top reviewers in the field. Secondly, the review process is generally more efficient from a time perspective, in that reviewers for top journals are generally given a maximum of 10 to 14 days to review a manuscript.

## Collaboration

International collaboration between higher educational institutions is indeed recommended in that it enhances the citation and hence quality of research outputs.3,29,30 However, collaboration creates a dilemma with regard to which individuals or higher educational institutions should be credited with research outputs generated by collaboration. If each country or institution involved is allocated a share of the collaborative publication (fractional counting) then the publication count will decrease.

Indeed, in a study of research outputs from Australian higher educational institutions, it was shown that collaboration resulted in a 17.8% reduction in the publication numbers for the period studied.3 Moreover, as the average citations per publication were greater in those publications involving collaborators (5.53 vs 4.22), the total number of citations assigned to Australia decreased by 24.5% for the period studied.3

In the South African context, only those researchers affiliated to a South African higher educational institution are credited by the Department of Higher Education and Training. To translate this into research output units, below are some examples of the research output units generated by accredited journal publications with and without national or international collaborators:

• four authors all affiliated to one South African higher educational institution: 4 × 0.25 = 1 unit
• two authors affiliated to one South African higher educational institution and two authors affiliated to another South African higher educational institution: 2 × 0.25 = 0.5 unit to each South African higher educational institution.
• two authors affiliated to one South African higher educational institution and two authors affiliated to one non-South African higher educational institution: 2 × 0.25 = 0.5 unit to the South African higher educational institution.

Bearing in mind the above examples, it is clear that the NFF does not encourage collaboration with researchers who are not affiliated to a South African higher educational institution.21,26

## Percentage public expenditure on higher education as a percentage of GDP and the financial value of research units

Over the past decade (2001–2009) the public expenditure on higher education as a percentage of GDP has increased in most countries [e.g. Argentina: 0.8% in 2001 to 0.9% in 2005 to 1.1% in 2009; Brazil: 0.7% in 2001 to 0.8% in 2005 to 0.8% in 2009; Finland: 1.6% in 2001 to 1.7% in 2005 to 1.8% in 2009; France: 0.9% in 2001 to 1.1% in 2005 to 1.2% in 2009; Ghana: 0.8% in 2001 to 1.1% in 2005 to 1.3% in 2009; Rwanda (values for 2001 not available): 0.6% in 2005 to 1.1% in 2009; Spain: 0.9% in 2001 to 0.9% in 2005 to 1.0% in 2009; New Zealand: 0.9% in 2001 to 0.9% in 2005 to 1.1% in 2009; USA: 0.9% in 2001 to 1.0% in 2005 to 1.0% in 2009],17 although it has declined in some [Botswana (values for 2001 not available): 1.0% in 2005 to 0.9% in 2009; South Africa: 0.8% in 2001 to 0.8% in 2005 to 0.6% in 2009; United Kingdom: 0.8% in 2001 to 0.9% in 2005 to 0.4% in 2009].17

More specifically, in South Africa, the percentage of public expenditure on higher education as a percentage of GDP declined from 0.86% in 1987 to a value of 0.64% in 2008, with the greatest decline occurring between 1999 (0.80%) and 2002 (0.68%).15 Although similar declines have occurred in other countries (Australia: 1.50% in 1974/75 to 0.89% in 1997/98 to 0.8% in 2009; United Kingdom: 0.8% in 2001 to 0.4% in 2009),3,17 in South Africa the value of 0.74% in 2001 was well below the averages of 0.81% for a total of 84 countries worldwide; 0.85% for 15 other countries in Africa; 0.85% for six countries in South America; 0.88% for 13 countries in North America; and 0.95% for 21 countries in Europe.15

Moreover, the value in South Africa of 0.65% in 2007 was well below the percentages spent in sub-Saharan countries such as Botswana, Burundi, Ethiopia, Kenya, Lesotho, Ruanda, Senegal and Swaziland, where the values range up to 2.1% of GDP.31 Although the percentages in South Africa were predicted to increase to 0.68% in 2008/9, 0.71% in 2009/10 and 0.74% in 2010/11,20 the values recorded by UNESCO were 0.6% in 2007 and 0.6% 2009.17

The main impact of the decrease in government resources to higher educational institutions has been an increase in the number of students and an increase in tuition fees as a means of enhancing income generation.15 In most circumstances the increase in student numbers has not been accompanied by an increase in staff numbers; hence resulting in increments in the student-to-staff ratios and a consequent decline in research productivity. Indeed in South Africa, the rise in student-to-staff ratios from 12.7 in 1986 to 18.0 in 200315 was accompanied by an ~15% decline in publications from 1997 to 2003.15 In the historically advantaged institutions, the percentage decline in publications was even higher (20%).15

Comparable changes occurred at Australian universities when faced with a decrease in governmental financial support to higher education from 1.50% in 1974/75 to 0.89% in 1997/98.3 The student-to-staff ratios rose from 12.81 in 1990 to 17.81 in 1999.32 However, despite the increased demands placed on staff, the publication output increased by 25% over this time period.3 The unexpected increment in publication output was primarily attributed to the introduction of a system to distribute funds to higher educational institutions in Australia based on publication outputs.3 However, the negative impact of this system was a decline in the quality of the publications (see section titled ‘Quantity versus quality’).

## Financial value of research outputs

Between 1987 and 2003, when the SAPSE formula was in use, the government subsidy awarded per publication unit was on average ~R22 000.21 With the introduction of the NFF in 2005, the block grants awarded to higher educational institutions for research were stopped; hence resulting in ~R1.5 billion becoming available for rewarding research output on a competitive basis.21 Consequently, the value of one research output unit rose from R77 606 in 2005 to R85 023 in 2007,19,21 and then to R102 604 in 2009.21 However, the value appears to have stabilised in more recent years, R110 000 in 201133 and currently R119 331, hence lending some credit to the criticism that the awarding of research funding using the NFF is a ‘zero-sum’ game.21,22

In comparison, in Australia the monetary value of a publication increased from A$761 (R6 664.31) to A$1 089 (R9 536.71) between 1995 and 2000.13 Furthermore, as a consequence of increases in the total amounts to be distributed by government, this value subsequently increased to A$3 000 (R26 271.94) in 2002.13 Other examples (obtained online) of the financial value of publications include:

• Qatar University: for publications in journals with an impact factor > 1.0, QR3 000 (R6 995.53) is awarded (40% to main author and 60% divided equally among co-authors including the main author) for each impact factor.
• University of South Pacific, Fiji: F$4 000 (R18 915.48) for A* (top) journals, F$3 000 (R14 186.61) for A journals, F$2000 (R9 457.74) for B journals and F$1 000 (R4 728.87) for C journals. The A, B, C, D ranking is according to the Australian Business Deans Council’s rankings, which is used in preference to the journal impact factors in order to account for the variation in impact factors across disciplines. The awards are given as part of the researchers’ salary and are taxed.
• Universities in Finland: FIM80 000 (R161 862.08) per impact factor is awarded to the institution rather than the individual.

## Distribution of Department of Higher Education and Training research subsidy within higher educational institutions in South Africa

## Financial reward of individuals versus institution

As the extent of governmental subsidy to higher educational institutions depends on the subsidy granted by the Department of Higher Education and Training for publications in accredited journals, higher educational institutions encourage academics to publish in accredited journals. However, at most of the higher educational institutions, authors only receive a proportion of the total subsidy. In general, the higher educational institutions receive the major share of the funding, although this does differ between the different higher educational institutions (see section titled ‘Different policies within and between South African institutions’).

As discussed above, the policies also differ between higher educational institutions worldwide. For example, in Spain the individual researcher is the recipient of the rewards for research outputs,6 whereas in Finland it is the institution and not the individual who benefits.1 In Australia, similar to South Africa, the various higher educational institutions differ in the policies they have adopted.3,13 Some have chosen to reward the individual researcher primarily, whereas at others the institutions are the major beneficiary.3

## Different policies within and between South African institutions

What is provided below are some examples (those which are accessible) of the different policies adopted by the various higher educational institutions in order to distribute the subsidy block grants received from the Department of Higher Education and Training based on research outputs:

• Fifty per cent to faculty, of which 70% to individual researcher and 30% to faculty for publications in international journals, or of which 50% to researcher and 50% to faculty for publications in South African journals.
• Proportion of university research budget is distributed to each faculty based on the units produced by each of these faculties. Each faculty individual research committee decides on the disbursement to the schools. Schools then decide whether to distribute to departments, divisions and/or individual researchers. It is clearly stipulated that these funds may only be used for research-related or academic activities and that the funds may not be used to supplement salaries.
• Sixty per cent to the individual researcher, 15% to faculty or department, 15% to research office, 10% to vice chancellor’s office. It is clearly stipulated that these funds can only be used for research and research-related activities. No cash payments are made to individual researchers.
• The university research committee allocates to each faculty a proportion of the funds received in the annual block grant. The proportion awarded to each faculty is based on a formula which considers the accredited outputs of each faculty and the throughput of postgraduate students, and includes recognition of outputs from the arts (such as design, compositions, exhibitions and performances).
• One higher educational institution incentivises researchers by rewarding top researchers with annual research awards of up to R500 000. The recipients of these awards are allowed to keep half for personal use, but have to use the rest for their research. However, the exact source of funding for these research awards is not clear.

## Conclusions and proposals

As government policies on research funding of institutions have a direct impact on the behavior of academics with regard to research outputs, there is a need to refine these policies in order to produce the desired academic behaviours. To avoid the possibility of producing quantity at the expense of quality, an element of quality needs to be incorporated into the policy. A proposal, based on the Spanish system, would be to incorporate a higher weighting for publications in higher impact factor and/or rank in discipline journals. It is probable that such a system would also address the disincentive provided by the current formula, to collaborate internationally (as international collaboration generally results in increased quality of publications).

In addition, the potential perverse incentive created by the awarding of developmental grants to underperforming institutions could be minimised by substantially reducing the monetary value of the developmental grants. Moreover, developmental grants should not be awarded on a continual basis. In other words, an institution that receives a developmental grant has to show substantial annual improvements in order to warrant further developmental grants.

A bolder proposal, which would support the curtailment of developmental grants, is that more funds should be given to those higher educational institutions where most of the publications are produced, in order to ensure that more research will be done at these institutions. Many believe that a greater incentive to individuals would be provided if a larger proportion (although probably not all) of the subsidy earned was granted to the individual(s) who generated the subsidy. However, these funds should be used for research purposes and not for the supplementation of salaries (bearing in mind the tax implications). Importantly, a way forward is to take heed of the evidence provided and thereby avoid the mistakes made by some, and follow the successful examples of others.
